# ‘We expected more about sex in the sex week’ - A qualitative study about students’ experiences with a sexual health education programme, from a health-promotion perspective

**DOI:** 10.1080/17482631.2021.1963035

**Published:** 2021-08-10

**Authors:** Elin Helbekkmo, Helene Trengereid Tempero, Ragnhild Sollesnes, Eva Langeland

**Affiliations:** Department of Health and Caring Science, Faculty of Health and Social Sciences, Western Norway University of Applied Sciences, Bergen, Norway

**Keywords:** Adolescents, complicity, health promotion, positive sexuality, qualitative content analysis, salutogenesis, sense of coherence, sexual health, sexual health education

## Abstract

**Purpose:**

The purpose of this study is to explore adolescents’ experiences with participation in a sexual health education programme named «Week 6», from a health-promotion perspective.

**Methods:**

Six focus group interviews were conducted with adolescents aged 15–16 in Norway. Qualitative content analysis was used to analyse the data material.

**Results:**

The results can be summed up by the main theme: “We like «Week 6» but … we expected more about sex in the sex week”. The main theme consisted of two main analysis-derived themes: “The students want a topical sexual health education, with realistic and relevant learning subjects and exercises”, and “The students want to contribute to the content and implementation, in order to improve the learning outcomes of «Week 6»”. Further, four categories were identified: organization and content, positive experiences, the potential for improvement, and learning outcome. «Week 6» is desirable, but students expect to learn more. Teaching should have a positive approach, and adolescents do not want their educators to get embarrassed.

**Conclusion:**

Although the teaching methods with active participation are enjoyable, it is necessary with more time for discussions and questions. Student participation in planning and implementation of the programme seems crucial for promoting salutogenic teaching processes.

## Introduction

Good sexual health is considered a resource that promotes quality of life and contributes towards overall health and well-being in individuals (WHO, 2015a). The World Health Organization (WHO) contemplates sexual health education as a lifelong learning process of acquiring information and of forming attitudes, values, and beliefs about identity, intimacy, and relationships. Access to information and education relating to sexuality and sexual health is essential to enable people to promote their health and make informed decisions about their lives (WHO, 2015b). The purpose of sexual health education is to ensure that children and adolescents obtain the necessary expertise regarding conditions that promote coping, joy of life, and a sense of self-worth throughout life (Directorate of Health, 2019).

Previous research shows that sexual health education is criticized by young people. Sex is presented in a moralistic, heteronormative, and reproductive way. The focus is on preventing sexually transmitted diseases and unwanted pregnancies (Pound et al., 2016). This seems to be the situation in many countries. In the Netherlands, a forerunner when it comes to sexual health education, adolescents rate their education as mediocre (Cense et al., 2020). Today’s young people are exploring sexuality and identity on the internet. Pornography is available and should not be the only source of information on sexual pleasure (Eleuteri et al., 2017; Mattebo et al., 2014). Adolescents wish for topics related to sexual health education to convey a positive view of sexuality and diversity. They wish adults to accept that young people have and desire sexual experiences and want to talk about what and how sex is (Aranda et al., 2018; Berggrav, 2015; Pound et al., 2016). Further, research shows that education focusing only on abstinence is ineffective in promoting positive changes in sexual behaviour (Denford et al., 2017). Sexuality must be recognized as a special subject for the students and should not be taught like ordinary subjects (Pound et al., 2016). Sexual health education is successful when students participate, the environment feels safe, and the teacher has sufficient knowledge, uses humour, and is confident when talking about sexuality (Aranda et al., 2018; Denford et al., 2017; Mattebo et al., 2014; Pound et al., 2016). Peer-led interventions are considered a powerful tool in sexual health education regarding influencing students’ knowledge and attitudes (Sun et al., 2018).

In Norway, the programme «Week 6» is produced and owned by the organization Sex and Politics and is designed to meet different learning goals from the school’s curriculum about sexuality. It is called «Week 6» because the schools usually run the programme in February, in the sixth week of the year. The number six is also pronounced the same way as the word “sex” in the Norwegian language. Sex and Politics describes the programme as a validated material and a helpful tool in the school’s sexual health education. It is designed for usage in different age groups in the primary, lower, and upper secondary education. The purpose of sexual health education is to empower young people with information, knowledge, and necessary action skills to make choices and take control of their own sexual health (Directorate of Health, 2019). The Norwegian Directorate of Health (2019b) describes «Week 6» as an intervention and a tool that has a formative influence on sexuality in a public health perspective. The programme is constructed to be a helpful tool for teachers and school health nurses/services and consists of various exercises in which students are active participants. These exercises include finding solutions to different dilemmas and taking a stand on various claims. During «Week 6» the timetable is cleared of its normal content, and students learn about sexuality throughout the week. «Week 6» consists of the primary programme and an additional programme with a new theme every year. In 2019, the theme of the additional material was *positive sexuality*. Sex and Politics provides guidelines for using «Week 6», but it is up to the schools to decide how they use the materials.

To the best of our knowledge, there are no previous research describing sexual health programmes that are comparable to the programme «Week 6». Further, as far as we know, there are no prior studies that investigate students’ experiences as participants in a sexual health programme such as «Week 6». The aim of this study is therefore to explore the experiences of students who have participated in this programme. More specifically, the research question is: How do students, from a salutogenic health promotion perspective, experience their participation in the sexual health education programme «Week 6»?

## Theoretical framework

Sexual health is widely understood as a state of emotional, mental, physical, and social well-being when it comes to sexuality, encompassing not only certain aspects of reproductive health but also the opportunity to have pleasurable and safe sexual experiences (WHO, 2015a). “Health promotion” is defined by the World Health Organization [WHO] as a process through which individuals and groups can take control, master and improve their health (Ministry of Health and Care Services, 2016a). The salutogenic model is defined as a health promotion theory (Antonovsky, 1996). In this theory, health is seen as a resource on a continuum with different levels. Health promotion measures focus on a sense of coherence (SOC), which is an expression of the individual’s ability to find and mobilize resistance resources to cope with challenges. SOC is strengthened when life is meaningful, comprehensible, and manageable. To promote SOC, it is necessary to experience continuity, balance between overload and underload, and an opportunity to participate in decision-making regarding one’s own life (Antonovsky, 1987). The purpose of a salutogenic approach is to promote a positive interaction between resistance resources and SOC, so that when challenges in life are appropriately experienced, they contribute to a positive health pattern (Langeland & Vinje, 2013).

Good sexual health is a salutogenic resource and protective factor that might promote coping skills, identity, SOC, and quality of life (Antonovsky, 1987; Ministry of Health and Care Services, 2016a). The theory of salutogenesis will therefore be used as a theoretical framework in the present study.

## Method

### Research design

This qualitative study has a phenomenological-hermeneutic research design, in accordance with Graneheim et al. (2017). Through an inductive approach, our aim was to explore the adolescents’ experiences with «Week 6» from a health-promotion perspective.

### Sample

A total of 31 students in 10th grade in lower secondary school were strategically selected to participate in the study. The participants were recruited from two schools in two different municipalities in the southeast area of Norway. The sample contained 11 boys and 20 girls aged 15–16 from four different classes.

To be included, the school must have arranged «Week 6» multiple times before. Furthermore, the students must have participated in «Week 6» and attended the same school throughout secondary school.

### Data collection

Six semi-structured focus group interviews were conducted from October to December 2019. Four focus-group interviews from a school in southern Norway and two focus-group interviews from a school in eastern Norway were arranged. One of the schools arranged «Week 6» every year during lower secondary school, and the other arranged it only once during ninth grade. The interviews were conducted 8–10 months after the students’ participation in «Week 6». Our sample therefore encompassed both students who had received the programme once and students who had received it twice. Neither school used the additional material about positive sexuality.

The first authors were present in all the focus-group interviews. The groups consisted of 4–7 participants. The first interview was conducted as a pilot study. The pilot interview was included, as the interview guide needed only slight justification. During the interviews, the first authors cooperated in collecting data. In each interview, one had the role of moderator and was responsible for welcoming the students and leading the interview. The other had the role of co-moderator. Both asked questions, and they switched roles after each interview.

Open questions were asked during the interviews. The questions in the interview guide were organized based on respectively structure, process, and results, such as: “Can you tell us how the teaching was conducted?” “What was it like to participate in «Week 6»?” and “What do you think about «Week 6?” The interviews lasted approximately 60 minutes and were conducted at school during schooltime. The interviews were recorded with consent.

### Analysis

The purpose of the analysis was to organize and find meaning and structure in the data (Polit & Beck, 2017). This work started by aiming to achieve a good overview and to make verbatim transcriptions from the recordings after each interview. The material was then analysed, following the approach of Graneheim and Lundman (2004). In the qualitative content analysis, words, sentences, or paragraphs were collected in meaning units in each interview. The text in the meaning units was thereafter shortened down and coded. Then, the preliminary meaning units were analysed across participants and the developed final categories, subthemes, and an overall theme that safeguarded the participants’ experiences. All authors discussed the analysis process and agreed upon subcategories, categories, sub-themes, and the theme. See Table I.

**Table I.** Overview of the analysis from subcategories to theme

Subcategories	Categories	Subthemes	Theme
Extent of «Week 6»	Organization and content	The students want a topical sexual health education with realistic and relevant learning subjects and exercises.	We like «Week 6», but … “we expected more about sex in the sex week”.
Who teaches			
Classroom environment			
Content			
Different week	Positive experiences		
Active participation			
External educators			
Questions answered		The students want to contribute to the content and implementation to improve the learning outcomes of «Week 6».	
Relevance	Potential for improvement		
Teacher and teaching			
Organization of the week			
Increased knowledge	Learning outcome		
Reflection			
Classroom environment			

Two increase transferability and show the richness of the data, Table II shows two examples of the steps in the analysis from meaning unit to category.

**Table II.** Examples of the analysis process from meaning unit to category

Meaning unit	Code	Sub-category	Category
Donna: I thought maybe we should learn more about how it feels to have sex, not just about how it’s done, in a way.	More about sex	Relevance	Potential for improvement
Benjamin: I think it was different, and therefore it was a lot more fun, so, like, I wanted to do it, and I was a little more motivated to do it, and stuff like that. Right.	Got more motivated	Active participation	Positive experiences

### Ethical considerations

The Norwegian Centre for Research Data (NSD) approved the study in August 2019 (NSD 162002). The participants were given written information about the study and their rights, where it was specifically pointed out that they could withdraw from the study at any time without needing to provide an explanation. Both the adolescents and their caregivers gave written consent to participate in the study. The data were anonymized and stored confidentially. In addition, the school health nurse was informed of the study to safeguard the students’ wellbeing.

## Results

The results will be presented in one overall main theme, subthemes, and categories, following the structure of the analysis process shown in Table III.

**Table III.** Overview of the findings

Main theme	Subthemes	Categories
We like «Week 6», but … “we expected more about sex in the sex-week”	The students want a topical sexual health education, with realistic and relevant learning subjects and exercises	Organization and content
		Positive experiences
	The students want to contribute to the content and implementation, for better learning outcome in «Week 6»	
		Potential of improvement
		Learning outcome

**We like «Week 6», but … ‘we expected more about sex in the sex week’**

The students’ experiences with «Week 6» varied between the two schools included in our study. The students who received the sexual health education programme every year in secondary school were overall more satisfied with their experiences. The students who only had «Week 6» once during secondary school, however, reported overall dissatisfaction with the programme. All students entered the week with high expectations about their learning outcomes. They expected to gain deeper knowledge and wanted to learn as much as possible. Students found the week different and fun, but it did not fulfil most students’ expectations. Although «Week 6» is considered a good idea, the findings show that the students expected the week to be all about sex. The theme was formed based on the students’ overall experiences with «Week 6» as something they wanted but that did not turn out to be what they expected.

*Donna:* *I thought we were going to learn more about how it feels to have sex, not just, sort of, how you do it.*

Throughout the week, the students said they learned more about the negative aspects of sex than the positive. The focus was on preventing incidents such as sexual diseases and unwanted pregnancies. They felt that sex was presented as a technical matter, and that the contents failed to present desirable aspects of sexuality.

*Fiona:* *Sex is not supposed to be a negative thing. But they make it seem like that.*

*The students want a topical sexual health education, with realistic and relevant learning subjects and exercises*

It is of great importance for the students to get a sexual health education that is topical. The students reported that the contents of «Week 6» have to feel relevant and realistic. They want to learn about all aspects of sex, and, overall, more positivity towards sex, such as sexual pleasure. The students express their desire to obtain in-debt knowledge about the following subjects: sex, the positive sides of sex, pleasure, abusive words and language, sexual physiology, myths about sex, falling in love, sexual arousal, their own gender, and confidence. The course should contain exercises that reflect situations the students find realistic. The examples in the dilemmas could have been better, and the students want them to present scenarios they could experience in real life. To ensure this, they suggested contributing to making them.

*Hermine:* *(…) If you are in a difficult situation, you can’t always talk, you feel, sometimes you feel like you can’t talk to people about it. So, if you can discuss the difficult situations, then maybe it will be a little easier.*

*Lilly:* *Situations we might experience, that we don’t already know.*

### Organization and content

Both schools included in this study used the primary teaching material of «Week 6». Neither used the additional material for 2019 about positive sexuality. One school arranges «Week 6» every year of lower secondary school (three years). These students therefore participated in the programme for the second time. On their timetable, they had four hours of «Week 6» lectures every day. At the other school, the programme is only arranged in ninth grade. These students had «Week 6» lectures all day every day throughout the week. However, in addition to topics about sexuality, they learned about Sami people in their Norwegian subject.

Their teachers gave most of the lectures. Both schools were visited by external educators. At one school, they had visits from the organization Sex and Society (Sex and Society is Norway’s largest clinic for sexual and reproductive health and rights). The other school was visited by a priest, a sexologist, and the organization Amathea (Amathea is a nationwide health service that guides individuals in matters of unplanned pregnancy and abortion). The school health nurse had lectures as part of «Week 6» at both schools. To create a safe environment, they all had class rules to follow during the week. Some students got to participate in making these.

During the week, the students went through a variety of subjects like reproduction, genitals, contraceptives, sexually transmitted diseases, sexual orientation, falling in love, gender diversity, porn, abortion, identity, self-image, queer sexuality, and Sami people. Students at both schools reported that they also had various exercises during the week, some in which they had to find solutions to different dilemmas, practice the use of a condom, addressing anonymous questions, conversions in boy- and girl-groups, and taking a stand on various claims.

### Positive experiences

The students said «Week 6» felt like a different week compared to their ordinary weeks, and this was a positive experience. They enjoyed the week, and it made them feel more motivated to learn and eager to come to school.

*Alba:* *[It was an] alternative week, something other than just regular school. You get a little … like, you have a little fun together for a week and do different things instead of just subjects.*

Most students enjoyed being an active part of the learning process by attending the exercises throughout the week. Some students said it felt good to express their opinions, and some said it was exciting to listen to other students’ thoughts. This study reveals that the students enjoyed getting lectures from external educators, and the majority preferred these over the lectures given by their own teachers. The students felt the external educators were more enthusiastic when teaching, which made the students more engaged and motivated. They had more knowledge and expertise, and they did not get embarrassed during the lessons, which was of great importance to the students. This made the students more comfortable and less awkward. Lastly, the students felt that the external educators took their questions seriously. All the students agreed that it was of great importance to ask questions and get these questions answered.

*Irene:* *It was very nice to get answers to some of the things we had questions about. So that was okay, really.*

*The students want to contribute to the content and implementation of «Week 6» to improve the learning outcomes*

The students expressed a desire to be included in advance to contribute to the content of «Week 6». They also wanted to be heard in decisions during the week and to get the opportunity to express their opinions in an evaluation afterwards. It was a recurring wish among students to take part in order to ensure and improve the learning outcomes.

*Sarah:* *They should try to take our perspective into consideration, and not just focus on what they want to teach us, but what* we want *to learn.*

The students also want to be included in deciding time allocation during the week. They very much enjoyed being active during «Week 6» and had a desire for more time spent on discussions and addressing questions. They had many questions throughout the week and uttered a desire to get them all answered. The students experienced that there was not enough time for discussions. They did, for instance, not get to discuss their choices when they took stands in the different exercises.

### The potential for improvement

The students reported that some of the content of «Week 6» lacked relevance. The students who had a subject about Sami people during their sexual health education did not find this relevant. This had a big impact on these students’ recollection of the week and seems to be an important reason for dissatisfaction.

*Researcher:* *All in all, what do you think about «Week 6»?*

*Olivia:* *Bad. Really bad.*

*Rihanna:* *Not what I expected.*

*Olivia:* *We had been looking forward to this. Or I had, at least. I thought the programme would be much better.*

Some students reported that they did not learn enough due to many subjects being rushed through, both from their teachers and from the external educators. They also felt that during some lectures, their teachers read directly from a textbook and lacked knowledge about sexuality. While some students found it easier to address the subjects in «Week 6» with their teachers, with whom they already had a relationship, most students preferred the external educators. They reported that their teachers often got embarrassed and evasive when talking about sex-related topics. They did not want to answer questions, nor open up for discussions. The students pointed out that it is important to get their questions answered, even the foolish ones, and they need to be taken seriously.

*Rihanna:* *During that week I think they should take everything seriously.*

Some teachers refused to answer questions in class, and made the students write their questions down and put them in the question box to be answered later in the week. The students wanted their educators to elaborate and answer their question with more than one sentence. At one school, the school health nurse answered their questions. Unfortunately, she did not have enough time, nor did she know the answer to all the questions.

*Fiona:* *They [the teachers] would not answer our questions. They just said: “No, I don’t want to answer that”.*

The students reflected upon the educator’s reasons for not answering and thought that the reason could be that their teachers interact with them every day, got embarrassed or did not feel confident enough to answer them. This study finds that the educators need to be confident and have knowledge and expertise about the subjects they are teaching during «Week 6». It is of great importance that the teachers do not get embarrassed while talking about sexuality.

The students had some suggestions for improvements with regard to the organization of «Week 6». As the topics were very diverse, it would be better to finish one topic before starting a new one. The students who had «Week 6» only once during secondary school wanted to have it again next year. On a general basis, the students want more sexual health education. Some of them even suggested sexuality as a subject of its own.

*Isabella:* *You can for instance*, *never learn too much about sex. You cannot learn too much if it is a subject of its own.*

### Learning outcome

To the question about how the week had affected them, the students gave many different answers. Some said they learned that they were not alone in dealing with feelings and issues. While some students felt that they did not learn much, many students reported they had gained more knowledge. Others said the week must have contributed to increased knowledge, but they are unsure how. When thinking back, students mostly remembered the exercises they did in «Week 6» rather than the content of the subject. In addition to enjoying the exercises as an active learning process, they also thought of them as a useful way to prepare for real-life situations they might experience.

*Andrew:* *It was fun and a little different. And then you might need it later as well. At least when you hear others’ opinions too. If you ever get in such a situation.*

## Discussion

Although the experiences of «Week 6» varied among the students, they agreed that the concept is a good one. They liked that they get to actively participate and welcome «Week 6» as a pleasant change from regular school. This study highlights the importance for the students to get their questions answered. It is also necessary to have enough time for the students to learn in-depth knowledge, participate in exercises, discuss, and address questions. Students experienced that their teachers got embarrassed and elusive when talking about sex-related topics, and most students wanted to receive sexual health education from external educators. Furthermore, it was of great importance to the students that the teaching reflects realistic topics and situations that are of relevance to them. This study reveals that students expected to gain in-depth knowledge about sex in «Week 6», and also that it is important for students to be involved in the organization and implementation of «Week 6», and that they generally want more sexual health education throughout their schooling.

### Promoting participation, sexual health and SOC

During «Week 6», the students play an active part in the teaching process through various exercises. These exercises are a major part of «Week 6» and are highly appreciated by the students as a positive experience. Student participation has, in the previous research, been identified as a success factor for sexual health education (Denford et al., 2017). The students enjoyed the chance to express their opinions and to hear the opinions of their peers. This exchange of experiences makes the students feel they are not alone in dealing with their feelings and concerns. They even get to experience that some of their chaotic feelings are normal and it is not “just them” having concerns. They also felt that it was easier to address sex-related topics with their classmates after «Week 6». Adolescence is a transition period with many challenges. It is seen as a confusing time full of self-doubt, turbulence, and a feeling of exclusion, where young people try to find themselves. A sense of coherence (SOC) is especially being developed during adolescence and is promoted when life is meaningful, comprehensible, and manageable (Antonovsky, 1987). Ideally, young people should develop their understanding of reality through the awareness that their individual way of dealing with existence is a successful way. In the stressful period of youth, they learn to cope with their own health, and through a socialization process, they shape their experiences into a strong SOC (Antonovsky, 1987).

Although the students liked the teaching method of «Week 6», they thought that some of the dilemmas lacked relevance and could have been better chosen. Some students suggested that they could make dilemmas themselves and thereby practice on real-life situations they perceive to be realistic. This finding is compatible with the salutogenic theory, in which coping is promoted by a better understanding of one’s own situation and the use of different available and conscious resistance resources (Antonovsky, 1987). Practising coping through realistic dilemmas might be a useful resistant resource in their development of SOC.

There was a recurring desire among the students to participate in designing the contents of «Week 6». To promote SOC, it is necessary to have the opportunity to participate in decision-making regarding one’s own life (Antonovsky, 1987). To ensure and improve their learning outcomes, the students want to participate in order to learn more about the things they care about and feel curious about. They suggested having more time for discussion and addressing questions to meet their desire to exchange thoughts and opinions. This is supported by the Norwegian Children’s Commissioner [Barneombudet]’s (2018), who recommends involving adolescents in designing how sexual health education should be, and that they should be actively participating in the teaching process through discussions and conversations. By equipping young people with knowledge and an opportunity to participate in decision-making regarding their sexual health education, sexual health might be improved, thus possibly also develop and improve their SOC.

The «Week 6» material is intended to be a helpful tool in the school’s sexual health education. This study shows that there were variations in the sexual health education students received during «Week 6» between the two included schools. Although some suggestions for improvement refer to the content of the material, most of them refer to the school’s usage of the material. Some of the students’ dissatisfaction concerned lack of time for discussion during the exercises and not getting their questions answered. Each individual school has the freedom to choose to use the whole material or parts of it. However, the organization Sex and Politics provides some guidance for the usage of «Week 6» and recommends for instance, that the teacher reads through and, if necessary, checks up on academic content prior to answering questions. In guidelines describing the exercise dilemma, they are estimated to take approximately 10–30 minutes, depending on whether one or several dilemmas are raised (Sex and Politics, n.d.b). The students who participated in «Week 6» every year in their lower secondary school were overall more satisfied. This might be because they spent more time on exercises and questions that were of great importance to them, or to getting «Week 6» every year with all the opportunities the programme provides. They also seemed more relaxed about missing out on subjects this year and the lack of in-depth knowledge. This might be because they know they can address these subjects next year. This makes sense from a salutogenic point of view, as a state of health includes confidence that the world is experienced as understandable, structured, and predictable (Antonovsky, 1987).

### The health-promoting aspect of a positive approach to sexual health education

This study confirms that young people think sexual health education is important and meaningful. They want more sexual health education and are eager to learn and understand more about sexuality. Access to knowledge, education, and information about sexuality and sexual health is fundamental for young people to achieve coping skills and control over their own sexual health (Ministry of Health and Care Services, 2016a). This study emphasizes the importance of sexual health educators being able to talk about sexuality without getting embarrassed. Educators need to be confident and have enough knowledge and expertise when talking about sexuality. This is supported by the previous research (Denford et al., 2017; Mattebo et al., 2014; Pound et al., 2016). Though some of our participants said they wanted to be educated by their teachers, whom they already had a relationship with, the majority preferred getting educated by the external educators. External educators are more confident and are specialists in sexual health. They can offer students in-depth knowledge about their teaching subjects. And finally, they do not get embarrassed.

The study emphasizes the students’ desire to have their questions taken seriously and answered. Educators must elaborate and give answers in more than short sentences. The school health nurses enjoy a strong reputation among students who experience getting guidance in issues regarding sexual health (Directory of Health, 2019). It is therefore unfortunate that the school health nurse at one of the schools was among those who lacked knowledge and could not answer the student’s questions. In the context of improving sexual health education, teacher suitability is often mentioned. Given the recommendation that school health nurses be involved, it is necessary to ensure that they are suitably and sufficiently educated in sexual health as well. A synthesis of qualitative studies by Pound et al. (2016) on young people’s views on sexual health education states that sexuality is a special subject for students and should not be taught like other subjects. Our participants liked that «Week 6» was an alternative week compared to their usual ones. The students were more motivated, and the week felt more relaxed, due to the change in their timetable. «Week 6» is a good attempt to teach sexuality in a way that recognizes sexuality as a special and important subject for students.

The students want to learn about sexual health from a positive point of view. They felt the focus was mainly negative and neglected aspects of sexuality such as pleasure and enjoyment. This is also shown in other studies (Aranda et al., 2018; Denford et al., 2017; Mattebo et al., 2014; Pound et al., 2016). As one of our informants so precisely expressed it: “Sex is not supposed to be a negative thing. But they make it seem like it is.” This study shows that students desire information about positive sexual experiences, including pleasure, confidence, and falling in love. They desire in-depth knowledge about all aspects of sexual health, including sexual myths and physiology.

Understanding is a key dimension in the SOC concept, and SOC develops through coherent life experiences (Antonovsky, 1987). That «Week 6» is a pun on the word “sex” in the Norwegian language makes it a compelling name and makes it stand out from other school-based teaching programmes. This strong association between “6ʹ and ‘sex’ might contribute to the students” expectation that they should have learned more about sex than they actually did. As sexual health is seen as a resistance resource to cope with challenges, it is important for young people to acquire enough knowledge to make good choices regarding their own health. Gaining knowledge about the positive aspects of human sexuality helps protect against the negative aspects (Denford et al., 2017) and promotes well-being.

One of the aims in the Norwegian sexual health education has for a long time been to prevent sexually transmitted diseases and reduce unwanted pregnancies and abortions. Previous summary of research shows that students are dissatisfied with their sexual health education, and this has been remarkably constant over a period of 25 years (Pound et al., 2016). In Norway, the abortion rates have been dropping since 2008 and are now at its lowest point ever registered (Ministry of Health and Care Services, 2016a). Recent research from Finland has shown that intervention focusing on preventing sexually transmitted diseases is effective to improve better knowledge and more frequent testing for diseases (Pakarinen et al., 2020). One can therefore argue that even though students show dissatisfaction with received sexual health education, it had a desired effect. On the other hand, our findings show that sexual health education should pay attention to the student’s wishes and have a positive focus on sexuality. It should be acknowledged that sex is a special subject for students, as well as the fact and range of young people’s sexual activity. Our study shows the importance of ensuring student complicity in the planning and implementation of «Week 6». If received sexual health education lack relevance young people will disengage from it and opportunities for safeguarding and improving their sexual health will be reduced (Pound et al., 2016).

The schools need to recognize their responsibility for providing knowledge. If students do not perceive the school’s sexual health education as being relevant, they seek information elsewhere. Pornography is easily accessible online and is used by young people as a source of information and positive sexual experiences (Mattebo et al., 2014). The technological advances of the last decade have thus provided greater access to misleading information. Almost all Norwegian children aged 9–16 have access to a computer, and 98% of young people use the internet daily (Ministry of Health and Care Services, 2016b). Pornography and information on the internet should not be the only source of knowledge about sexual pleasure for young people. The Norwegian Directorate of Health (2019) states that sexual health education from confident adults with relevant competence is an important corrective and contribution. However, for some students, the only opportunity to learn about sexuality from confident and competent adults is at school. Primary school in Norway is compulsory. Therefore, from a health-promotion perspective, sexual health education in schools ensures that everyone gets the necessary information to improve their own health. Increasing young people’s life skills is important for sexual health. Provided with relevant information, young people know best themselves what they need in order to improve their own health (Ministry of Health and Care Services, 2016b).

Our study shows that students want a topical sexual health education. They want to talk about what sex is and how sex feels. They are simply calling for a positive approach to sex and sexuality. The same wish is well documented in the previous research (Aranda et al., 2018; Berggrav, 2015; Denford et al., 2017; Mattebo et al., 2014; Pound et al., 2016).

### Strengths and limitations

The strength of this study is that we have had four researchers throughout the entire process: preparing the study, data collection, analysis, and the writing process. This has contributed to comprehensive and critical reflections among the researchers. Our discussions revolved around interpretations of codes, sub-themes, main theme, as well as the study methods and results. Consequently, we have been conscious of our own preconceptions, which we know may affect the data. Our consciousness about this contributed to the dynamic process that is vital in qualitative research (Malterud, 2011). We have, to the best of our ability, allowed the data to speak for themselves. It has been important to describe and explore the students’ experiences respectfully to promote trustworthy knowledge. During the interviews, the students reported experiencing a safe environment, and we therefore believe they have answered to the best of their ability. One weakness of the study material is relying on the informants’ recollections. We interviewed them 8–10 months after they participated in «Week 6». However, having focus groups was a strength, allowing them to help each other remember during the conversations. It is also a strength that two schools were included in the sample. This has provided us the opportunity to study two different organizations and usage of the sexual health education material. This study has been a contribution to discovering students’ experiences with «Week 6», and it has been important for us to present our informants credibly.

Providing a detailed presentation of the findings and analytical procedure, we hope we have shown sufficient validity to facilitate transferability. It is reasonable to expect that students at other schools with comparable ways of organizing «Week 6» will have similar experiences. Our aim was to gain a deeper understanding of students’ experiences with the programme. As far as we know, this has been the first study to explore adolescents’ experiences with and views on this programme. Therefore, this study contributes to an increased understanding of the importance of involving students in the planning process of sexual health education programme «Week 6».

### Implications

One of the aims of Norwegian sexual health education has long been preventing sexually transmitted diseases and reducing unwanted pregnancies and abortions. Previous summaries of research show that students are dissatisfied with their sexual health education, and this has been remarkably constant over a period of 25 years (Pound et al., 2016). Our findings show that sexual health education should pay attention to the students’ wishes and have a positive focus on sexuality. It should be acknowledged that sex is a special subject for students, as should the fact and range of young people’s sexual activity. Our study shows that it is important to ensure student involvement in the planning and implementation of «Week 6». If received sexual health education lacks relevance, young people will disengage, and opportunities for safeguarding and improving their sexual health will be reduced (Pound et al., 2016). In this study, we have explored students’ experiences with «Week 6» primary material from Sex and Politics. Even though their experiences vary, all the students included in this study would recommend it to other students. Sex and Politics have made a great contribution to improving sexual health education with this material. Future research should aim to study students’ experiences with receiving the additional material about positive sexuality, as our study indicates a need for a positive approach. This study shows that students appreciate the teaching methods in «Week 6». It would therefore be interesting if someone surveyed whether this additional material has taken into consideration the students’ criticisms regarding lack of positive focus in received sexual health education. The present study indicates that we need several studies that explore what is needed to implement a good programme for sexual education where educators can ensure the students’ active participation.

## Conclusions

This study has highlighted that it is essential to include students in the planning of and decision process for «Week 6» to enhance their learning outcomes. The teaching methods in «Week 6» are good. However, the students want more time for discussion and addressing questions during the week. An important health-promoting factor is to enable the students to make good choices regarding sexual health by providing them with knowledge and skills.

The way we see it, there have been enough studies that verify the health-promoting aspect of a positive approach to sexual health education. Thus, the time has come for the school’s sexual health education to be the source of information for young people on positive sexual experiences. Studies on how to best implement this are needed. Not the least, we need more studies on students’ experiences with «Week 6» to get a broader picture of students’ experiences with the programme.
